# Assessment of Resilience Training for Hospital Employees in the Era of COVID-19

**DOI:** 10.1001/jamanetworkopen.2022.20677

**Published:** 2022-07-01

**Authors:** Joyce P. Yi-Frazier, Maeve B. O’Donnell, Elizabeth A. Adhikari, Chuan Zhou, Miranda C. Bradford, Samantha Garcia-Perez, Kelly J. Shipman, Samantha E. Hurtado, Courtney C. Junkins, Alison O’Daffer, Abby R. Rosenberg

**Affiliations:** 1 Palliative Care and Resilience Lab, Seattle Children’s Research Institute, Seattle, Washington; 2 Cambia Palliative Care Center of Excellence, University of Washington, Seattle; 3 Center for Child Health, Behavior, and Development, Seattle Children’s Research Institute, Seattle, Washington; 4 Division of General Pediatrics, Department of Pediatrics, University of Washington School of Medicine, Seattle; 5 Biostatistics, Epidemiology, and Analytics in Research Program, Seattle Children’s Research Institute, Seattle, Washington; 6 Division of Hematology/Oncology, Department of Pediatrics, University of Washington School of Medicine, Seattle

## Abstract

**Importance:**

Health care workers face serious mental health challenges as a result of ongoing work stress. The COVID-19 pandemic exacerbated that stress, resulting in high rates of anxiety, depression, and burnout. To date, few evidence-based programs targeting mental health outcomes in health care workers have been described.

**Objective:**

To assess the feasibility, acceptability, and preliminary outcomes of a skills-based coaching program designed to reduce stress and build resilience.

**Design, Setting, and Participants:**

A pilot cohort study was conducted between September 2020 and April 2021 using preprogram and postprogram assessments and a mixed-methods analysis. Duration of follow-up was 7 weeks. The coaching program was delivered via video conferencing. Participants were health care workers and staff from a large urban health system.

**Intervention:**

The Promoting Resilience in Stress Management (PRISM) program, a manualized, skills-based coaching program originally developed for adolescents and young adults with serious/chronic illness, was adapted to support health care workers and staff (“PRISM at Work”). It included 6 weekly 1-hour group sessions.

**Main Outcomes and Measures:**

Feasibility was defined a priori as 70% completion rates. Acceptability was defined quantitatively (satisfaction scores) and qualitatively (open-ended questions regarding experience with program). Preliminary outcomes were assessed with preprogram and post program assessments of self-reported resilience, stress, anxiety, and burnout using validated instruments. Descriptive statistics summarized demographic variables and feasibility and acceptability. Linear mixed effects regression models examined preliminary outcomes, controlling for relevant covariates.

**Results:**

A total of 153 participants (median [SD] age, 40.6 [10.1] years; 142 [92%] were female; 128 [84%] identified as having White race; 81 [53%] were in patient-facing roles) enrolled. Of the 132 health care workers who provided follow-up surveys, 120 (91%) had completed the program, and 116 (88%) reported being satisfied. Answers to open-ended questions suggested that participants wanted more PRISM either with longer or additional sessions. Participant-reported resilience (β = 1.74; 95% CI, 1.00-2.48), stress (β = −2.40; 95% CI, −3.28 to −1.51), anxiety (β = −2.04; 95% CI, −2.74 to −1.34), and burnout-exhaustion (β = −0.37; 95% CI, −0.56 to −0.18) improved after the program.

**Conclusions and Relevance:**

Results of this study suggest that PRISM at Work may have utility for health care workers and staff in that the program was found to be feasible, acceptable, and associated with improved outcomes.

## Key Points

**Question:**

Is a group-coaching program designed to reduce stress and teach resilience skills feasible, acceptable, and preliminarily useful for health care workers during the COVID-19 pandemic?

**Findings:**

In this pilot cohort study of 153 health care workers and hospital employees from a single health system, the group-coaching program was feasible and acceptable, as demonstrated by high demand, retention, and satisfaction. Measured according to validated scales, self-reported resilience, stress, anxiety, and burnout improved among participants.

**Meaning:**

Results of this study suggest that resilience-building programs may support improved mental health outcomes.

## Teaser

This cohort study assesses the feasibility and acceptability of a skills-based training program for health care workers and hospital employees during the COVID-19 pandemic as well as preliminary outcomes as indicated by participant-reported resilience, anxiety, stress, and burnout.

## Introduction

Frontline health care workers (HCWs) and hospital staff face serious mental health challenges as a result of ongoing work stress and burden.^1^ Substantial numbers of physicians, nurses, and medical staff report experiencing burnout and emotional exhaustion.^1,2,3,4^ The intense and unpredictable demands of the COVID-19 pandemic quickly led to further mental health consequences for this already at-risk group.^5,6,7,8,9,10^ Systematic reviews cite high prevalence of burnout, stress, sleep disturbances, and other anxiety symptoms in HCWs during the pandemic.^11,12^ The mental health of HCWs has become a vital concern in the infrastructure of the US health care system.^13,14^

To date, few evidenced-based methods have addressed burnout and poor mental health among HCWs. We postulated that a specific focus on bolstering resilience, defined as the process of harnessing resources to maintain well-being in the face of substantial stress,^15^ would be an important factor for HCWs particularly during the pandemic. The Promoting Resilience in Stress Management (PRISM) program is an empirically designed skills-based training program positioned to mitigate stress and build resilience in groups facing elevated levels of stress.^16,17,18^ Previous work translated this patient-centered program to one that supported HCWs (“PRISM at Work”).^19^

This study was designed to determine the feasibility and acceptability of PRISM at Work for HCWs and hospital employees during the COVID-19 pandemic. We also examined preliminary outcomes by assessing changes in participant-reported resilience, anxiety, stress, and burnout. We hypothesized that HCWs would find PRISM to be feasible, acceptable, and helpful for navigating the stress and burdens of their daily work and lives.

## Methods

### PRISM at Work Program

PRISM is a manualized (ie, associated with strict protocols for fidelity of delivery) skills-based training program originally developed for adolescents and young adults with serious or chronic illness. With a theoretical basis in stress and coping theory, PRISM intentionally diverged from traditional cognitive behavioral therapy by focusing instead on brief skills-based training for practicality and feasibility.^18^ The program was rigorously tested and refined with stakeholder feedback from patients and families, and feasibility, acceptability, and efficacy have been demonstrated in groups of patients with varying diseases.^16,17,18,20,21^ In response to COVID-19, we translated and piloted PRISM to a program that could also support health care staff.^19^

### Content

PRISM at Work is composed of a 6-session group-based curriculum administered by 2 trained PRISM coaches on a video conferencing platform. The program included 6 weekly 1-hour sessions, covering (1) science of resilience, (2) stress management, (3) goal setting, (4) cognitive reframing, (5) meaning-making, and (6) coming together and moving forward with resilience. Sessions include didactic teaching of the skill, group reflection, and discussion of next steps in smaller breakout rooms. Participants in this study also received access to an award-winning smartphone app,^22^ which facilitated participants’ practice and tracking of skills, and handouts describing each skill and how to practice the techniques. Details of the program have been reported in previous publications.^16,18,19,20^

### Delivery and Fidelity

Four nonmedical research staff were trained to facilitate group sessions. These coaches included individuals with master’s and doctorate level psychology or social work degrees, consistent with the training of coaches from other PRISM trials.^16,18,20^ Each coach completed 6 to 8 hours of training, which included supervised leading of pilot groups, practice scenarios, and review of the script. Audio for each class was recorded to assess fidelity to the script.

### Participants and Recruitment

Eligible participants (HCWs and staff) were initially recruited from a children’s hospital, and the program was extended to nursing staff at the affiliated University Medical Center. Courses were advertised via institutional announcements and through division and hospital leadership. Attempts to minimize bias related to availability were addressed by offering courses on various times and days. All interested participants provided signed informed consent. Enrollment occurred from September 2020 to April 2021. We delivered 2 to 3 PRISM courses in 5 waves, every 6 to 8 weeks. At study start, 12 participants at maximum were enrolled for each course; based on participant feedback, we reduced class sizes to 10 midway through the study. This study was approved by the Seattle Children’s Hospital Institutional Review Board. Data reporting followed the Strengthening the Reporting of Observational Studies in Epidemiology (STROBE) reporting guideline for cohort studies.

### Procedures

Participants completed presurveys prior to the first session and within 1 week after the final session, for a total follow-up period of 7 weeks. Completion of the presurvey was required to participate in the first session, and attendance in the first class was required to proceed in the program. To optimize future implementation, we examined participant comments throughout the course of the study using an iterative approach, with the intent of making content changes as necessary. Participants were considered active as long as they attended the first class and did not explicitly ask to be withdrawn.

### Primary Outcomes

Our primary aims were to assess feasibility and acceptability. Feasibility was assessed per previous studies^18,20^ by several authors of the present study and defined a priori by 70% completion among enrolled participants. We also observed the number of filled classes and the average number of participants on waitlists. PRISM coach fidelity was assessed as part of feasibility, including adherence to the script, which was reviewed and confirmed by coaches immediately after each session. In addition, we monitored the number of psychosocial or emotional concerns that would warrant additional intervention or support.

Acceptability was defined quantitatively and qualitatively. Three questions assessed acceptability using a 5-point Likert scale. The first was “How satisfied were you with the PRISM program?” The second was “How satisfied were you with the frequency of the sessions?” The third was “How satisfied were you with the length of the sessions?” As part of the postsurvey, acceptability was also assessed with 3 open-ended questions: “Who do you think would benefit from this type of program?”; “What did you like best about the program?”; and “What suggestions do you have for the program?”

Analysis of preliminary outcome data was determined by preprogram and postprogram assessments with validated instruments. Self-perceived resilience was assessed with the 10-item Connor-Davidson Resilience scale.^23,24^ Items are scored on a 5-point Likert scale (range 10-40); higher scores reflect higher resilience. Adult population norms are: 25th percentile: 29 points, 50th percentile: 32 points, 75th percentile: 36 points.^24^

Stress was assessed with the 10-item Perceived Stress Scale, a widely used global stress measure assessing the degree to which life situations are stressful.^25^ Items are scored on a 5-point Likert scale; higher scores indicate higher stress. Internal consistency, reliability, and validity are established.^26,27^ Scores ranging from 0 to 13 indicate low stress, from 14 to 26, moderate stress, and from 27 to 40, high stress.^26^

Anxiety was assessed with the Generalized Anxiety Disorder 7-item scale (GAD-7)^28^; items are scored on a 4-point Likert scale with higher scores indicating higher anxiety. The GAD-7 has good reliability, and validity, with cut points of 5 interpreted as mild anxiety, 10 as moderate anxiety, and 15 as severe anxiety.^28^

Burnout was assessed with the 16-item Maslach Burnout Inventory (MBI)–General Survey.^29,30^ Total scores and cut points are not recommended^29,30^; instead the scale includes 3 subscales: exhaustion (feelings of being overextended and exhausted by one's work), cynicism (an indifference or a distant attitude toward one’s work), and professional efficacy (satisfaction with past and present accomplishments). Published mean (SD) norms for raw scores in health care were for exhaustion, 2.2 (1.5), for cynicism, 1.4 (1.2), and for professional efficacy, 4.2 (1.0).^29,30^ The MBI-General Survey has been used with diverse populations of health care workers, including nurses, laboratory technicians, and hospital managers.^31,32^

Hope was assessed with a 12-item Hope Scale, measuring overall perception that one’s goals can be met.^33^ The Hope Scale includes a total score and 2 subscales: pathway, indicating one’s perceived ability to generate a route to their goals, and agency, indicating one’s perceived ability to initiate and maintain the actions necessary to reach a goal. Strong internal reliability and validity have been established.^33^ Items are scored on an 8-item Likert scale with higher scores indicating higher hope.

Benefit-finding was assessed with an adapted 17-item Benefit-Finding Scale with each item expressing potential benefit from the COVID-19 experience.^34,35^ Items are scored on a 5-point Likert scale; higher scores indicate more benefit-finding. This scale has demonstrated internal consistency, reliability, and validity in multiple cancer studies.^35,36^ For use in the current study, items remained the same; the question stem was changed from “having had breast cancer” to “the COVID-19 pandemic.”

Meaning in work was assessed with the 10-item Work and Meaning Inventory, assessing different dimensions of experiencing work as meaningful. Items map onto 3 factors: positive meaning from work, meaning-making through work, and greater good motivations.^37^ Items are scored on a 5-point Likert scale. This scale is reliable, valid, and highly related to work-related and general well-being variables.^37^ Higher scores indicate higher meaning.

Demographic and work-related variables were collected at baseline including self-reported gender, age, language, education, marital status, and race and ethnicity. Job category was determined by binary categorization of their job title: patient-facing (interacted regularly with patients/families) and nonpatient facing (did not interact regularly with patients/families). Two additional variables were coded for each participant as covariates: cohort indicating the individual class they participated in and wave indicating the specific week in which their class began.

### Statistical Analysis

Participant-reported demographic and work-related variables, feasibility data (percent who completed the program, attendance, and rates of sign-up), and acceptability data (ie, percent satisfied) were summarized descriptively using numbers and percentages. For qualitative analysis of acceptability data, the Standards for Reporting Qualitative Research (SRQR) were followed to code responses.^38^ Four coders (M.B.O., E.A.A., S.G P., and S.E.H.) conducted directed content analysis with Dedoose qualitative analysis software, version 7.0.23 (SocioCultural Research Consultants LLC) using an a priori codebook based on questions from the interview guide and informed by previous PRISM trials.^39^ Once the codebook was finalized, the same coders sequentially coded each transcript into code categories. Consensus meetings refined codes and resolved discrepancies. Numbers of similar responses within a code category were tabulated and divided by the number of total responses, yielding a percentage of endorsement for that item.

A sample size of 115 was determined to provide 80% power to detect completion rate greater than 70% if an actual 80% completion rate with 2-sided α .05 type I error was observed. Outcomes were analyzed using linear mixed-effects regression models controlling for age, job category, marital status, and group order of training (wave). These variables were chosen for the possibility that certain age groups, patient-facing jobs, family composition/support, and different phases of the pandemic might be associated with higher stress levels and increased response to the training. A nested random effects structure was included in all models to account for clustering due to repeated assessments within participants and participants receiving training in group setting. All analyses were performed using the R statistical software, version 4.1.0 (R Foundation for Statistical Computing). Statistical significance was set at 2-sided *P* = .05.

## Results

### Study Population

A total of 153 participants provided informed consent with a mean (SD) age of 40.6 (10.1) years; 140 (92%) were female; 12 (7%) were male; 1 (1%) was nonbinary; 8 (5%) were Asian or Asian American; 4 (8%) were multiracial or biracial; 2 (1%) were Hispanic; and 128 (87%) were White (Table 1). A total of 81 participants (53%) were in patient-facing roles (ie, nurses, physicians, allied health professionals) and 72 (47%) in nonpatient facing roles (eg, managers, research staff, business offices).

**Table 1.** Characteristics of PRISM at Work Participants

Variable	No. (%)
Total participants	153 (100)
Gender	
Female	140 (92)
Male	12 (7)
Nonbinary	1 (1)
Age, y	
Mean (SD)	40.6 (10.1)
<30	24 (16)
30-39	55 (36)
40-49	36 (23)
50-64	30 (20)
≥65	2 (1)
Missing	6 (4)
Race and ethnicity^a^	
Asian or Asian American	8 (5)
Hispanic, Latino, or Latinx	2 (1)
Multiracial or biracial	8 (4)
Pacific Islander or Native American	1 (1)
White	128 (84)
Missing	6 (1)
Primary language	
English	148 (96)
Punjabi	1 (1)
Ukrainian	1 (1)
Chinese	1 (1)
Missing	2 (1)
Job category	
Patient facing	81 (53)
Physicians (MDs)	8 (5)
Nurses or advanced practice clinicians	49 (32)
Other (ie, social work, nutritionists, family support)	24 (16)
Nonpatient facing (ie, research, management, administration)	70 (46)
Missing	2 (1)
Level of education	
High school graduate	1 (1)
Some college, no degree	6 (4)
Associate degree	3 (2)
Bachelor's degree (ex, BA, AB, BS, BBA)	72 (47)
Master's degree (ex, MA, MS, MEng, Med, MBA)	53 (34)
Professional school degree (ex, MD, DDS, DVM, JD)	9 (6)
Doctoral degree (ex, PhD, EdD)	9 (6)
Marital or cohabitating status^b^	
Married	89 (58)
Divorced	11 (7)
Separated	4 (3)
Never married	32 (21)
Living with partner	16 (10)
Missing	6 (4)

^a^ Race and ethnicity are presented as categorized by patient self-report.

^b^ Could choose more than one option.

Baseline instrument scores suggested low levels of self-perceived resilience (Connor-Davidson Resilience scale score, mean [SD],27.0 [5.2]), moderate level of stress (Perceived Stress Scale score, mean [SD], 18.4 [5.9]), moderate level of anxiety (GAD-7 scale score, mean [SD], 7.8 [4.7]), and high levels of burnout-exhaustion (MBI Exhaustion subscale score, mean [SD], 3.6 [1.3]) (Table 2).

**Table 2.** Association of Employee-Reported Outcomes and the PRISM at Work Program

Outcome	Overall summary statistics, mean (SD)		Regression results^a^								
	Baseline	Post-PRISM	Overall			Clinical/patient facing			Nonclinical/nonpatient facing^a^		
			β (95% CI)	*P* value	β (95% CI)		*P* value	β (95% CI)		*P* value
Resilience	26.9 (5.2)	28.7 (4.9)	1.74 (1.00 to 2.48)	<.001	1.54 (0.51 to 2.57)		.004	1.94 (0.88 to 3.00)		<.001
Stress	18.4 (5.7)	16.1 (6.0)	−2.40 (−3.28 to −1.51)	<.001	−2.05 (−3.24 to -.85)		.001	−2.73 (−4.03 to −1.42)		<.001
Anxiety	7.5 (4.6)	5.6 (4.3)	−2.04 (−2.74 to −1.34)	<.001	−2.08 (−2.80 to −1.35)		<.001	−2.00 (−3.20 to −0.79)		.002
Burnout-exhaustion	3.5 (1.3)	3.2 (1.4)	−0.37 (−0.56 to −0.18)	<.001	−0.45 (−0.73 to −0.18)		.002	−0.29 (−0.55 to −0.03)		.03
Burnout-cynicism	2.4 (1.5)	2.2 (1.5)	−0.22 (−0.41 to −0.03)	.02	−0.29 (−0.59 to 0.01)		.06	−0.17 (−0.41 to −0.07)		.17
Burnout-professional efficacy	4.5 (0.9)	4.6 (1.0)	.09 (−0.04 to 0.23)	.18	0.16 (−0.06 to 0.38)		.15	0.04 (−0.13 to 0.21)		.64
Benefit-finding	47.0 (11.5)	52.7 (13.3)	5.42 (3.59 to 7.25)	<.001	6.48 (3.92 to 9.04)		<.001	4.35 (1.75 to 6.94)		.002
Hope-total	41.5 (7.7)	43.4 (7.5)	1.85 (0.93 to 2.78)	<.001	2.05 (0.83 to 3.27)		.001	1.62 (0.21 to 3.04)		.03
Hope-agency	20.8 (4.4)	21.8 (4.2)	0.84 (0.32 to 1.36)	.002	0.68 (0.03 to 1.33)		.04	0.99 (0.17 to 1.81)		.02
Hope-pathway	20.5 (4.2)	21.6 (3.8)	1.03 (0.47 to 1.60)	<.001	1.37 (0.62 to 2.12)		<.001	0.65 (−0.19 to 1.49)		.13
Meaning in work	39.7 (6.3)	39.9 (7.0)	0.42 (−0.47 to 1.31)	.35	0.84 (−0.22 to 1.90)		.12	−0.02 (−1.45 to 1.42)		.98
Positive meaning	16.0 (2.9)	16.3 (2.9)	0.42 (0.01 to 0.84)	.04	0.41 (−0.09 to 0.91)		.11	0.44 (−0.23 to 1.10)		.20
Meaning-making	11.3 (2.0)	11.4 (2.4)	0.16 (−0.16 to 0.49)	.33	0.33 (−0.10 to 0.75)		.13	−0.01 (−0.51 to 0.48)		.96
Greater good	12.5 (2.3)	12.4 (2.3)	−0.07 (−0.40 to 0.26)	.69	0.10 (−0.36 to 0.56)		.67	−0.25 (−0.73 to 0.23)		.30

^a^ Based on linear mixed effects regression models with preprogram and postprogram indicator as the factor of interest (β). Age (continuous), job category (binary, patient facing vs nonpatient facing), marital status (binary, married or living with partner vs otherwise), and wave of program (integer, 1-5) were entered into the model as fixed-effects covariates. Participants and class wave were entered as nested random effects to account for clustering. Regression results also shown stratified by job category.

### Feasibility

We created 15 cohorts (individual classes) consisting of 8 to 12 participants comprising 5 waves (periods of time in which they started the course). Of the 153 who completed baseline surveys, 145 (95%) attended the first session. Of those who attended the first session, 113 (78%) attended 5 or 6 of the 6 total sessions. One-hundred percent of offered courses filled to capacity, with an average of 5 people on the waitlist per class. A total of 132 participants (91%) completed follow-up surveys, and 93 participants (64%) completed open-ended questions. The 21 participants (14%) who missed the follow-up assessment were not substantially different from the participants who completed the follow-up assessment in any of the demographic variables and baseline outcome values.

### Coach Fidelity

All 4 PRISM coaches successfully delivered the program at least once. Content fidelity was 100%.

### Observed Psychosocial or Emotional Concerns

We observed no psychosocial or emotional concerns associated with the PRISM at Work program. Specifically, no participant verbalized psychosocial or emotional concerns to a degree that prompted external support outside that of the program coaches.

### Acceptability

Of 132 HCWs who provided follow-up surveys, 116 participants (88%) reported being satisfied with PRISM overall, 120 participants (91%) were satisfied or very satisfied with the frequency of sessions, and 110 participants (83%) were satisfied or very satisfied with the length of sessions (Table 3).

**Table 3.** Acceptability of PRISM at Work for 132 Participants Who Completed Postprogram Questionnaires

Variable	No. (%)					
	Very satisfied	Satisfied	Neither satisfied nor dissatisfied	Dissatisfied	Very dissatisfied	Did not respond
Overall satisfaction with PRISM at Work	70 (53)	46 (35)	11 (8)	1 (1)	0	4 (3)
Satisfaction with frequency of sessions	76 (58)	44 (33)	6 (5)	1 (1)	0	5 (4)
Satisfaction with length of sessions	60 (45)	50 (38)	9 (7)	2 (2)	0	11 (8)

Abbreviation: PRISM, Promoting Resilience in Stress Management program.

Of the 92 participants who responded to the open-ended questions (Table 4), 91 participants (99%) were able to nominate someone at work who they thought would benefit from the program, and 44 (48%) stated that everyone in the workplace would benefit. When asked what they liked best, participants most often mentioned aspects of community, connection, and support (49% of responses). In addition, when asked how PRISM could improve, the most common suggestion was to increase the amount of time in the program, either by lengthening the sessions themselves or having more sessions altogether, with 24 (26%) respondents suggesting one of these options.

**Table 4.** Example Quotations From Open-Ended Acceptability Questions

Question	Example quotation [role]
Who do you think would benefit from this type of program?	“Everyone would benefit from this program. Conducting the training is a small group setting with phenomenal trainers also contributed to gaining a sense of community, in addition to the specific skills.” [physician]
	“I believe that first responders would benefit from PRISM bringing coping skills and learn ways to relax during these ongoing issues in the world. Anyone who has been working throughout the pandemic and has been isolated to working from home.” [researcher]
	“Our group had individuals from many different departments and each seemed to be having strong positive experiences with the program. I can only speak from a clinical perspective, but I think every level of staff in a patient care environment would benefit. I don't think it should be limited by job description though.” [physician]
What did you like best about the program?	“I liked getting to learn about other people's stories and experiences with resilience. I also learned a lot of techniques that I believe will be effective when it comes to incorporating them into my own life.” [researcher]
	“I really liked the group format of PRISM and actual tangible skills that were taught. I also really enjoyed the break out rooms–very helpful to talk to a smaller group of individuals to talk through our plans and how we were thinking about the skills.” [social worker/behavioral health care worker]
	“The breakout groups with our peers. It was really interesting to come together with different people and talk about goal setting and negative self talk and realize that we shared a lot of similar feelings and challenges around that work.” [IRB/regulatory affairs]
	“Love the app, love the session recaps, love the introduction and get people talking parts.” [RN nursing educator]
	“I loved the PRISM app and daily reminders to check in with myself re: stress/resilience.” [clinical services manager]
What suggestions do you have for the program?	“It sometimes felt like we could use another 15 to 30 minutes and I wonder if the sessions could be 75 or 90 minutes. All the sessions were excellent and really helpful.” [researcher]
	“Would prefer more practice of the tools in the sessions, especially the breathing. Overall, the balance is tipped too far to conceptual discussion and there was not enough practice and deepening of the tools.” [communications specialist]
	“The majority of my stress at work comes from systemic inadequacies and feeling extremely frustrated and alone. I’d like to explore tools to help address these systemic issues and how to ensure my voice is being heard and keep pushing forward.” [quality assurance specialist]
	“Future sessions also (could) include other critical skills (self advocacy, effective communication within hierarchical systems, systems-based change).” [physician]

Abbreviations: IRB, institutional review board; PRISM, Promoting Resilience in Stress Management program; RN, registered nurse.

### Preliminary Outcomes

Regression analyses controlled for age, wave of program, marital status, and job category. Given that participants were predominantly White and female, those variables were not used as covariates, and educational level was not used because of its potential association with job category. Regression results suggested that employee-reported outcomes were associated with PRISM at Work (Table 2). Specifically, resilience increased by a mean of 1.74 points (95% CI, 1.00-2.48 points), stress decreased by a mean of 2.40 points (95% CI, −3.28 to −1.51 points), anxiety decreased by a mean of 2.04 points (95% CI, −2.74 to −1.34 points), burnout-exhaustion decreased by a mean of 0.37 points (95% CI, −0.56 to −0.18 points), benefit finding increased by 5.42 points (95% CI, 3.59-7.25 points), and hope increased by a mean of 1.85 points (95% CI, 0.93-2.78 points). Results were similar for patient-facing and nonpatient facing roles (Table 2).

## Discussion

The PRISM at Work program was designed to help HCWs and hospital staff manage stress and improve resilience through a manualized, skills-based coaching curriculum. In this pilot cohort study conducted during the COVID-19 pandemic, we found PRISM at Work to be feasible and acceptable among those who agreed to participate. Our data also suggest that receipt of PRISM was associated with increased perceptions of resilience and reduced feelings of anxiety, stress and burnout from preprogram to postprogram assessments.

Given the nature of the COVID-19 pandemic, resilience resources for HCW and medical staff are needed. Although programs such as PRISM may not address systemic factors associated with burnout for health care workers and staff including long work hours, staffing shortages, and ongoing personal risk, and mandated programs may be less-likely to be well received, HCW well-being certainly can and should be addressed.^4,40^ Resilience-building programs have been consistently recommended for the workplace, both prepandemic and postpandemic, particularly for high-stress occupations such as health care.^41,42^ Existing guidelines for mental health programs for HCWs recommend easily accessible programs that are brief, simple, and incorporate digital components to augment convenience and accessibility.^43^ Facilitators to successful implementation of these programs include a positive, safe learning environment that includes formal and informal discussions and that provides opportunities for reflection and shared learning.^44^ PRISM at Work meets all of these recommendations.

For HCWs with high levels of job strain, attention to well-being and resilience is critical.^4,40,45^ In the era of COVID-19, resilience is a vital component in coping with ongoing stress, finding meaning in one’s work, and the future of the US health care system’s ability to weather burnout and adequately staff personnel.^46^ We found that mean resilience scores in this population (both preprogram and postprogram) were low in comparison with adult norms. It is unclear whether this finding is a manifestation of the COVID-19 pandemic or whether this low perception of one’s own resilience was common before the pandemic. Regardless, opportunities to learn, reflect on, and develop one’s resilience resources are necessary. These tools are not always intuitive or innate. We and others have shown that via deliberate action, individual resilience resources can be bolstered.^16,17,18,20,21^ In health care, it is important to note that such deliberate actions must come from both individual HCWs and the health care organizations who support them.^47^

### Limitations

This study has limitations. First, this was a pilot study which limited our ability to offer the course on a larger scale. Successful enrollment was based on motivation, self-selection, and the ability to attend the class. As a result, participants may have been more receptive to the content of this program. Further, staff had to take their own time to participate in this program, and thus we may have inadvertently created barriers to attendance. The number of physician attendees, for example, was limited. Similarly, those who responded to the open-ended questions may have been more favorable about the program than those who did not reply. Next, the demographic characteristics of participants indicated a lack of diversity. Although efforts were made to improve diversity by reaching out to the division heads and representatives for underrepresented groups, it remains unclear whether PRISM at Work would be feasible, acceptable, or preliminarily efficacious for staff who identify as men, nonbinary, or who have been historically marginalized. Future work must not only improve on the diversity of participants but also ensure that all eligible HCWs and staff have the opportunity to participate. Similarly, our experience at a single health-system may not be generalizable to other institutions.

With only 2 data points from each participant, we were limited in our ability to infer differences within participants. Change scores, although substantial, did not often cross cut-off thresholds. The design of this study as a cohort study limits our ability to infer efficacy. Larger trials may aid in confirmation of our preliminary findings.

## Conclusions

In this pilot cohort study, we found that the PRISM at Work program may be a promising resource with utility for teaching resilience skills to HCWs and hospital staff, a group facing elevated stress and poor outcomes. With growing attention and interest in improving these outcomes, results of our study suggest that integrating a resilience-promoting curriculum is feasible and acceptable. Further, use of the program was associated with improved self-reported resilience, stress, and burnout. PRISM at Work appears to be one approach toward offering deliberate action in response to prevalence of poor mental health outcomes in this community.
